# Inferring Physical Function From Wearable Activity Monitors: Analysis of Free-Living Activity Data From Patients With Knee Osteoarthritis

**DOI:** 10.2196/11315

**Published:** 2018-12-18

**Authors:** Vibhu Agarwal, Matthew Smuck, Christy Tomkins-Lane, Nigam H Shah

**Affiliations:** 1 Center for Biomedical Informatics Research Stanford University Stanford, CA United States; 2 Stanford University Hospital and Clinics Stanford, CA United States; 3 Department of Health and Physical Education Mount Royal University Calgary, AB Canada

**Keywords:** physical function, passive monitoring, physical function profile, wearable activity data, statistical learning

## Abstract

**Background:**

Clinical assessments for physical function do not objectively quantify routine daily activities. Wearable activity monitors (WAMs) enable objective measurement of daily activities, but it remains unclear how these map to clinically measured physical function measures.

**Objective:**

This study aims to derive a representation of physical function from daily measurements of free-living activity obtained through a WAM. In addition, we evaluate our derived measure against objectively measured function using an ordinal classification setup.

**Methods:**

We defined function profiles representing average time spent in a set of pattern classes over consecutive days. We constructed a function profile using minute-level activity data from a WAM available from the Osteoarthritis Initiative. Using the function profile as input, we trained statistical models that classified subjects into quartiles of objective measurements of physical function as measured through the 400-m walk test, 20-m walk test, and 5 times sit-stand test. Furthermore, we evaluated model performance on held-out data.

**Results:**

The function profile derived from minute-level activity data can accurately predict physical performance as measured through clinical assessments. Using held-out data, the Goodman-Kruskal Gamma statistic obtained in classifying performance values in the first quartile, interquartile range, and the fourth quartile was 0.62, 0.53, and 0.51 for the 400-m walk, 20-m walk, and 5 times sit-stand tests, respectively.

**Conclusions:**

Function profiles accurately represent physical function, as demonstrated by the relationship between the profiles and clinically measured physical performance. The estimation of physical performance through function profiles derived from free-living activity data may enable remote functional monitoring of patients.

## Introduction

Physical function is an important indicator of physiological well-being. Recently, physical status has become an outcome of interest in most medical specialties [1-4] and is increasingly regarded as the “sixth vital sign” [5]. Attempts at arresting and managing the functional decline must start with an evaluation of the baseline functional status. For example, maximizing improvement in advanced osteoarthritis requires knowing a patient’s baseline function to detect any improvement. Therefore, valid metrics to monitor physical function are necessary [6-8]. The International Classification of Functioning, Disability and Health [9] characterizes physical function in 2 distinct categories—capacity and performance. Capacity is the capability of a person to complete a given task in a controlled environment (eg, a timed walking test or a sit-stand test), while performance is what a person does in his or her current environment (eg, real-life physical activity monitoring). Traditionally, physical performance is estimated by surveys and self-reported questionnaires. One example is the assessment of one’s ability to complete the daily activities necessary to live independently (including bathing, dressing, toileting, transferring, maintaining bowel and bladder continence, and feeding), collectively referred to as the activities of daily living (ADLs) [10,11] and are typically measured by surveys. Disability indexes based on ADLs can differentiate healthy aging patients, patients with mild cognitive impairment, and patients with dementia [12]. However, ADL scores may have a response bias from self-reporting and low sensitivity to changes in high-functioning older adults [13]. In contrast, physical capacity measures (such as walking and sit-to-stand speeds and grip strength observed under supervision) capture variation across a wider range of physical function, including initial changes in the early stages of decline [14-16]. The main drawback of such capacity measures is that they require substantial time and effort from patients and researchers, as well as access to specialized facilities. The relationship between physical activity and physical function measures is a topic of active research [17-21].

Although the need to measure physical function is widely appreciated, self-reported assessments of physical performance are inadequate owing to poor discrimination and biases and difficulties in recalling historical activities; physical capacity measures require adherence to specific test protocols and are usually limited to research settings. Seeking a more simple and accurate measure, we have created a novel method for inferring physical function based on objective measurements of daily physical activity obtained from a wearable device. Our work enables quantitative monitoring of physical function—the first step toward improved precision in clinical research and practice.

Wearable activity monitors (WAMs), typically equipped with one or more accelerometers, provide a convenient way to measure physical activity objectively [22-24]. However, attempts to use WAMs to link the measured physical activity and physical function have been limited by their reliance on traditional methods of analyzing WAM data [25-28]. Two research groups [20,29] have demonstrated that the measured physical activity and physical capacity are associated but independent domains of physical function. For example, a change in physical capacity (eg, on the 6-minute walk test) need not imply a corresponding change in real-life activity levels. Interestingly, both research groups concluded that differentiating physical activity into classes leads to a stronger association with physical function, compared with a univariate measure based on average acceleration. WAMs typically measure the aggregate velocity change over a period—which by itself was considered inadequate for distinguishing classes of activities. We hypothesized that higher-order patterns in daily activity recorded by a WAM would correlate with physical function. We defined pattern classes from daily activity data using an unsupervised approach and used this information to create a *function profile*, which represents the mean allocation of time to different pattern classes. Using machine learning techniques, we classified activity profiles into discrete quartiles of commonly used measures of physical function such as the 400-m walk test (400MWT).

Studies with WAMs have, thus far, focused on the following:

1. Evaluation of measurement reliability and validity and characterizing activity phenotypes by patterns in free-living activity data [20,30-33].
2. Developing models of isolated activities and postures using supervised learning [34-37].
3. Developing activity-based models of physical capacity wherein subjects undergo instrumented versions of various capacity tests as summarized in a recent literature review by Grimm and Bolink [38].

**Figure 1:**
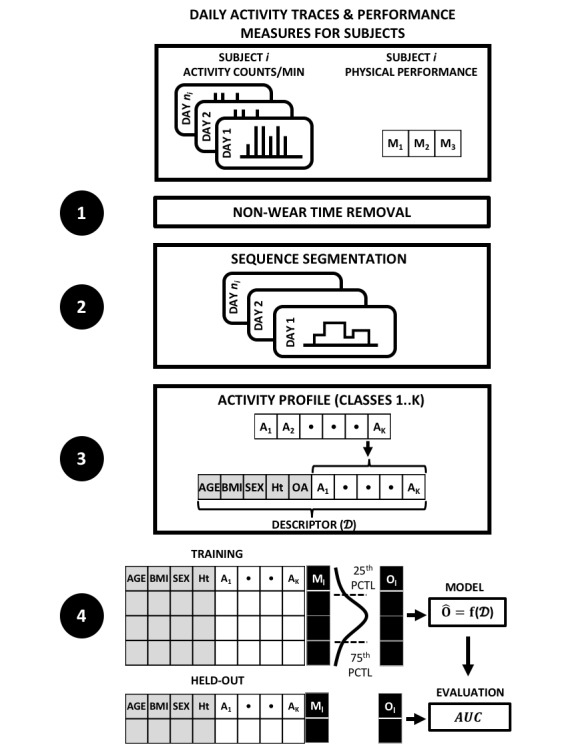
Estimating physical function from daily activity traces (overall workflow). Ht: height; OA: osteoarthritis; PCTL: percentile; AUC: area under the receiver operating characteristic curve.

Furthermore, Gresham et al used daily activity metrics (steps, distance, and stairs) to compute correlations with the clinically measured performance status in patients with advanced cancer [39]. In a study on nursing home residents, Merilahti et al reported an association between features derived from daily free-living activity and patient-reported physical function [40]. However, none of the studies mentioned above has modeled physical function using daily free-living activity—a crucial step in medical applications that require passive monitoring of function. Our research contribution is to use WAM data to characterize daily free-living activity into pattern classes and infer physical function based on the pattern classes. This study demonstrates the feasibility of distinguishing physical function categories with high sensitivity and specificity, and discusses potential uses in medical research and treatment. Figure 1 illustrates our overall workflow. Daily activity, measured as counts per minute, was recorded for 2001 subjects in year 4 of the OAI study. For each subject, various objective measurements were obtained from which we selected results for the 400-m walk test (400MWT), average pace on the 20-m walk test (20MPACE), and 5 sit-stand time (5CS), labeled as M1, M2, and M3 in Figure 1. Thereafter, nonwear time was excluded from activity traces, daily activity count sequences were segmented, and a composite feature descriptor with a daily activity profile was constructed for each subject. Finally, quantitative response values were converted to ordinal values based on empirical quantiles obtained from the training partition and a classifier for the feature descriptor was trained on the training partition of the feature matrix (80%) and evaluated on the held-out partition.

## Methods

### Data

We used publicly available data from the Osteoarthritis Initiative, (OAI) which follows a cohort of subjects who either had a clinical diagnosis (progression subcohort) of oseoarthritis or were at risk at baseline (incidence subcohort). The OAI has daily accelerometer measurements for subjects who participated in a physical activity study; these participants were instructed to use an ActiGraph GT1M uniaxial accelerometer (ActiGraph; Pensacola, FL, USA) continuously for up to 7 consecutive days, except during sleep and water activities. The ActiGraph GT1M is a compact, hip-worn device that measures dynamic acceleration in the range of 0.05-2.0 g; its validity and reliability have been established previously [41-43]. Participants maintained a daily log of water and cycling activities, as the accelerometer may not have been able to capture these accurately. A post facto analysis revealed that participants spent little time in these activities (median 0 minutes/day; interquartile range 0.0-3.4 minutes/day), indicating that little activity was missed by the monitors. Table 1 summarizes the key attributes of the physical activity study data (Multimedia Appendix 1).

The accelerometer data in OAI consist of activity counts per minute. An activity count is a weighted sum of discretely-sampled (30 Hz) values of one-dimensional acceleration. We used established guidelines to determine the wear time and valid days of activity monitoring, as reported previously [44]. Since 0 or low values of activity counts could also arise from nonwear time, we excluded nonwear periods. Continuous runs of 0 counts for >90 minutes (allowing for interruptions of up to 2 consecutive minutes with <100 counts) were discarded as nonwear periods. A day with a wear time of, at least, 10 hours was considered valid. Furthermore, objective, as well as patient-reported measures of physical function, were recorded during patient follow-up visits.

### Objective Measures of Function

The Osteoarthritis Research Society International [45] recommends testing of activities that are typically affected by OA. We selected 3 OAI performance measures that had equivalents in Osteoarthritis Research Society International’s recommended tests, which were as follows: the 400MWT, for which longer completion times are associated with a higher risk of mobility limitation and disability (adjusted hazard ratios, 4.43, *P*<.001), as well as a higher risk of death (adjusted hazard ratio, 3.23, *P*<.001) for subjects in the highest quartile [46]; the average pace in a 20-m walk test (20MPACE) is the closest available short walk-length evaluation in the OAI dataset that is used for gait speed assessment; the number of sit-to-stands per second measured over 5 repetitions (5CSPACE) as a measure of the sit-to-stand function, which has good test-retest reliability [47].

### Relationship With Daily Activity

Physical function is defined as the repertoire and relative proportion of activities that a subject accomplishes in a given environment. We recovered segments representing homogenous activities from the daily sequences of counts per minute obtained from a WAM and defined *pattern classes* based on similar segments. A subject’s function profile was computed as average minutes allocated to each pattern class. Finally, we inferred mappings from the function profile to the objective measurements of the function described in the earlier section using supervised learning.

#### Pattern Classes and Function Profile

**Figure 2:**
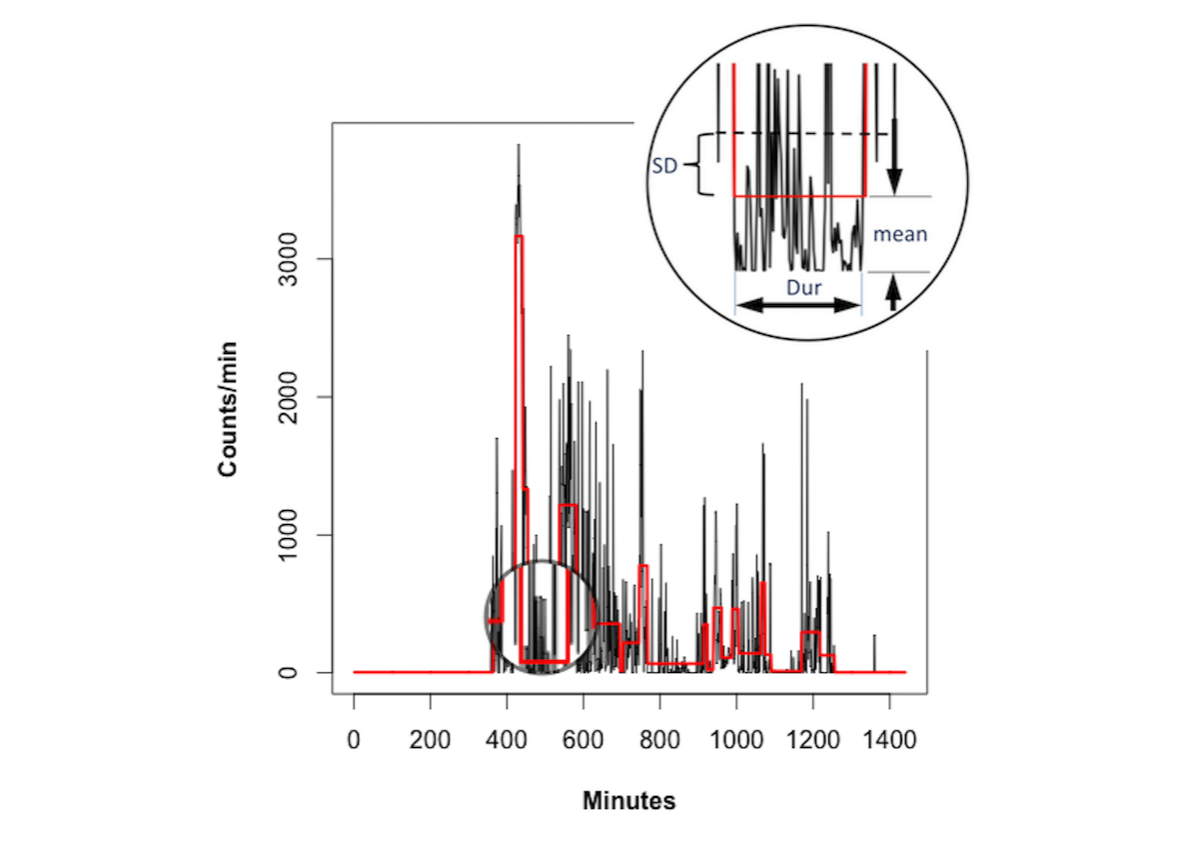
Segmentation of counts-per-minute sequences. Dur: duration.

We used the change-point analysis algorithm by James and Matteson [48] to segment counts-per-minute sequences; this algorithm searches for segment boundaries such that each segment represents a change in the distribution of the time-ordered counts with respect to preceding and subsequent segments. Figure 2 illustrates a counts-per-minute sequence for a typical subject on a given day and the segments that are recovered through change-point analysis (as shown by the horizontal red lines). Each segment is an instance of a pattern class whose mean and SD are estimated by the sample mean and SD of the segment.

Each segment was indexed using the mean and SD of the counts-per-minute values; this representation improves discrimination between classes of activity patterns (henceforth referred to as *pattern classes*) [49].

**Figure 3:**
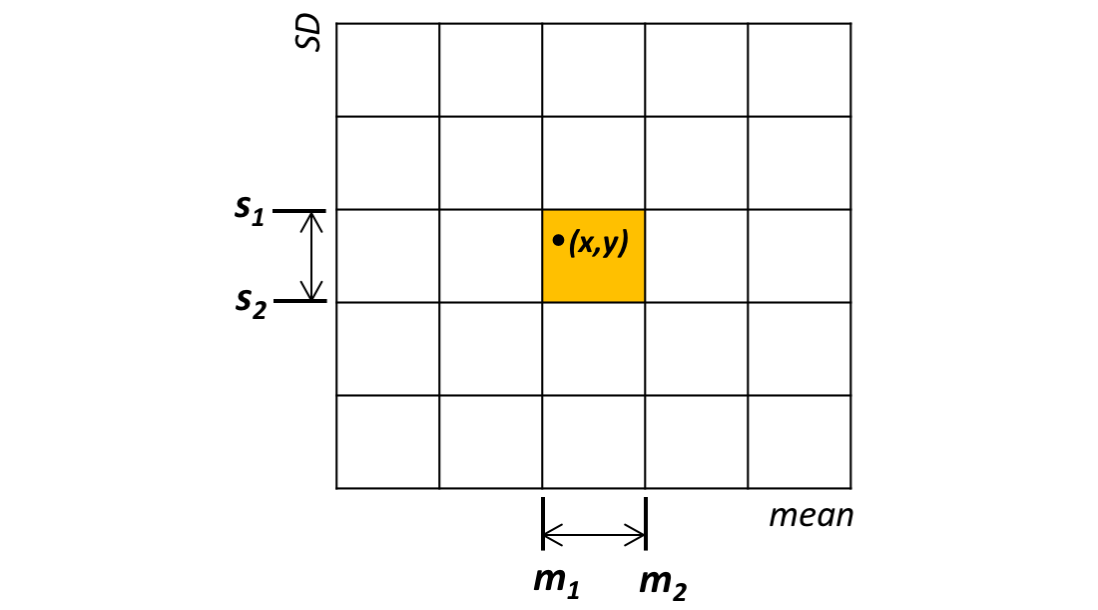
The mean and SD space containing all segments is partitioned into bounding regions, each defined by a mean and an SD interval.

A *pattern class* is a bounded region in the segment feature space. Our feature space *F* consists of all (*m, s*) vectors: *m* ∈[0, M], *s* ∈[0, S], where M and S are the maximum mean and SD over all segments found through the segmentation. A pattern class is defined by a pair of intervals such as [*m*_*1*_*, m*_*2*_) X [*s*_*1*_*, s*_*2*_). A segment with mean *x* and SD *y* (*m*_*1*_*≤x<m*_*2*_*, s*_*1*_*≤y<s*_*2*_) is an instance of the pattern class so defined. Figure 3 illustrates such a segment represented in the mean-SD space spanned by all segments and its assignment to a pattern class [*m*_*1*_*, m*_*2*_) X [*s*_*1*_*, s*_*2*_), as shown by the shaded region. Based on the pattern classes obtained from partitioning *F*, we defined a *function profile* for each subject as the average time allocated to each pattern class per day. The function profile for a subject *i* is given by

Ai=(a_i1_, a_i2_...a_iJ_) where

*a*_*il*_=(1/*K*_*i*_)∑_k_*t*_*ilk*_

*J:* the number of pattern classes

*k=1...K*_*i,*_ the number of days of observations for subject *i*

*t*_*ijk*_*:* the number of minutes spent by subject *i* in pattern class *j,* on day *k*

**Figure 4:**
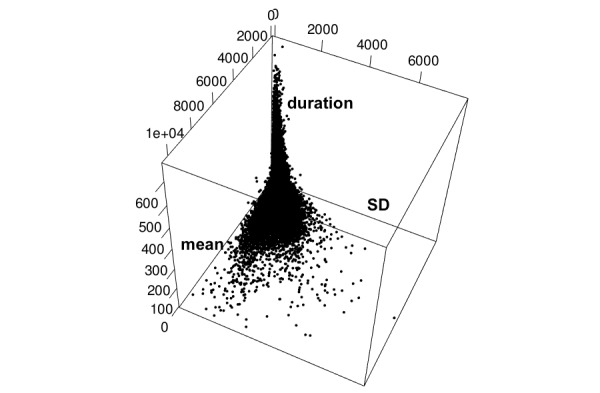
Scatter plot of the mean, SD, and duration of the segments.

As seen in Figure 4, the number of instances of a pattern class decrease as the mean and SD increase resulting in a sparse daily activity profile. *D*_*i*_*=(BMI*_*i*_*, Age*_*i*_*, Sex*_*i*_*, Height*_*i*_*, OA*_*i*_*, A*_*i*_*)*

**Table 1:** Key attributes of the knee osteoarthritis subjects providing physical activity data.

Characteristics		Value
**Total number of subjects (N=2001), n (%)**		
	Incidence subcohort	1490 (74.46)
	Progression subcohort	505 (25.24)
	Control subcohort	6 (0.30)
Gender (male), n (%)		891 (44.53)
Body mass index, mean (SD)		28.52 (4.87)
Mean Comorbidity Index		0.52
Median days of activity		7

#### Supervised Learning

We defined a composite descriptor *D*_*i*_*=(BMI*_*i*_*, Age*_*i*_*, Sex*_*i*_*, Height*_*i*_*, OA*_*i*_*, A*_*i*_*)* for each subject *i* in our data, where *OA_i_* refers to a subject’s baseline status (healthy, at-risk, or progressive disease) and *A*_*i*_ is the function profile. A regression function *f* (*D*) that maps *D*_*i*_ to an objective measure of physical capacity can be obtained by minimizing the expected squared error loss.

Medical studies commonly group continuous variables into quantiles for ease of interpretation and analysis [46,50,51]. We, therefore, defined our response variable by grouping the objective measure of physical capacity into ordered categories 1<2<3. As shown in Figure 1, categories 1 and 3 represented values in the lowest and highest quartiles, respectively, and 2 represented values spanning the interquartile range for a specific response. Classes 1 and 3 correspond to the upper and lower quartiles on the physical capacity measurements and, therefore, contain only half as many observations as in class 2. To address the imbalance, each observation was weighted by its class prevalence in the fitting procedure.

Generalized Additive Models (GAM) can identify and characterize nonlinear regression effects through an additive specification of nonparametric functions of the predictors. We used GAMs because fits from quantitative regressions suggest that at higher values, linearity in the predictors may not be a justifiable modeling assumption (Multimedia Appendix 2). A GAM may be specified as follows:


*g*(
*µ*(
*X*)) =
*α* +
*f*_1_(
*X*_1_) +
*f*_2_(
*X*_2_) +...+
*f*_p_(
*X*_p_) where

*µ*(
*X*) denotes the conditional mean of the response, that is, E[Y|X]

*g*(
*µ*(.) is the link function

*f*_1_...
*f
_p_* are the unspecified smooth functions for each of the p predictors

**Figure 5:**
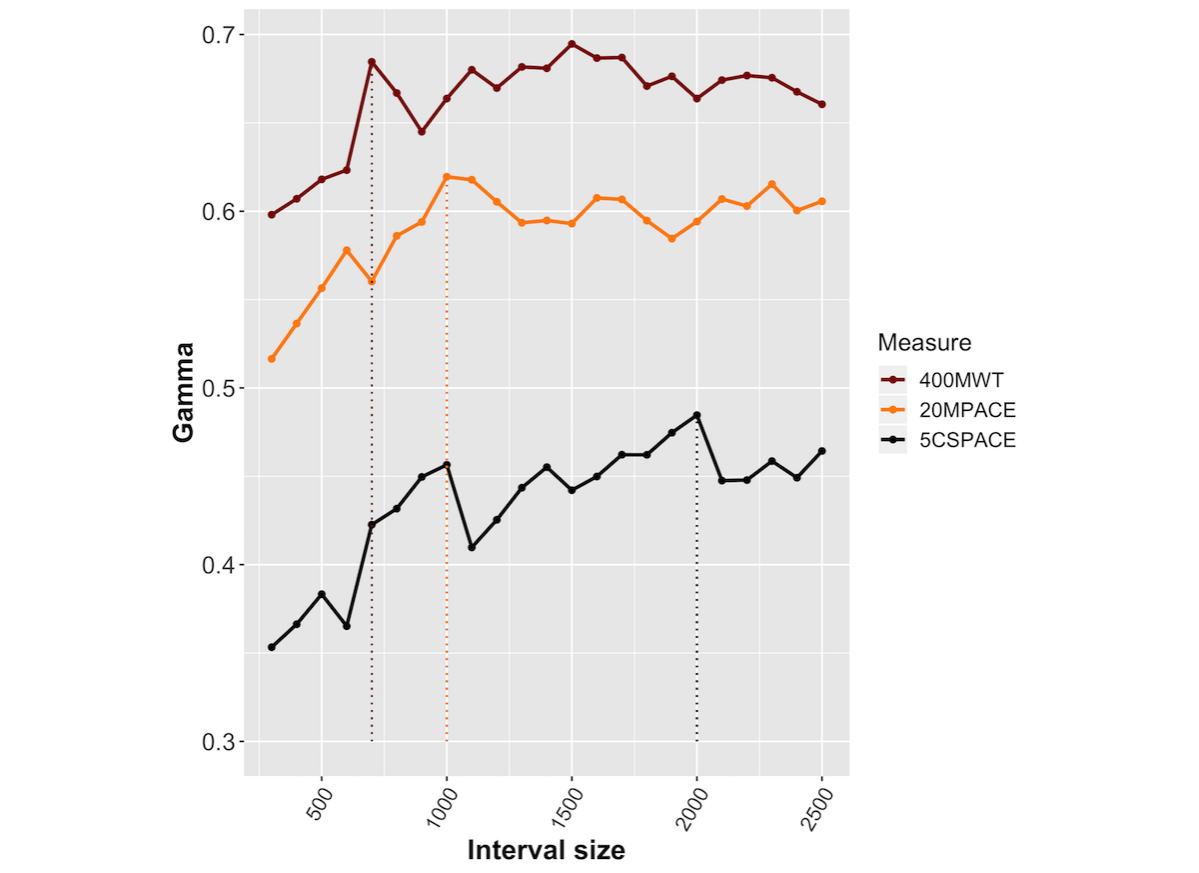
The mean Gamma (Goodman-Kruskal rank correlation between the predicted and true responses) in 5-fold cross-validation for 20MPACE (20-m walk test), 5CSPACE (number of sit-to-stands per second measured over 5 repetitions), and 400MWT (400-m walk test) models.

Unspecified functions of predictors are smoothers (typically kernels or cubic splines) that are estimated simultaneously using a backfitting algorithm [52]. The estimated reveal the nature of the predictor-response relationship. The function profile depends on the pattern classes, which are defined as intervals in the mean-SD space covering all segments. The size of the 2D interval in feature space that defines our pattern classes is a tuning parameter. Small intervals allow instances from adjacent classes to be in close proximity, increasing the correlation between activity profile elements. We ascertained the optimal size of the 2D interval—with equal mean and SD intervals—through repeated 5-fold cross-validation on our training data, as shown in Figure 5 (dashed lines indicate the optimal region size). Our intuition for the different optimal size for the 5CS model is that daily activities that involve sit-stand transitions are subsumed in the function classes defined on wider intervals. For example, sit-stand-walk and walk-stand-sit (measured by per minute activity counts) are transitions to and from activities characterized by large mean counts, whereas sit-stand and stand-sit are transitions to and from low mean count activities. On the other hand, most daily activities require some level of lower extremity strength, balance and gait initiation, and control capability—each of which are necessary for walking. Therefore, it seems reasonable that a profile constructed from function classes that distinguish between such activities will have a high correlation with walking test results.

We evaluated cross-validation performance using the mean Goodman-Kruskal Gamma [53], which measures the rank correlation between the true and predicted categories (Multimedia Appendix 2). For optimal bin sizes, GAMs were refit using the full training data and features based on the optimal bin size, and ordered categorical for the response family using the mgcv package [54]. We evaluated the Goodman-Kruskal Gamma for the predicted and true classes, using the held-out data.

## Results

### Principal Results

As described in the Methods section, we found homogeneous segments from daily activity sequences of counts per minute and defined pattern classes based on similar segments. A subject’s function profile was average daily minutes allocated to each pattern class. Finally, we learned mappings from the function profile to the objective measurements of physical capacity. Table 2 summarizes classifier performance for the GAMs on the held-out data using the function profiles based on the optimal interval sizes. The values in parentheses indicate improvement over baseline performance without function profile predictors.

Including the activity profile improved the held-out Gamma by 4%-10%, compared with classifiers in which the activity profile was excluded from the predictors, with higher improvement in classification of walking test results (Multimedia Appendix 3).

### Predictors of Physical Function

**Figure 6:**
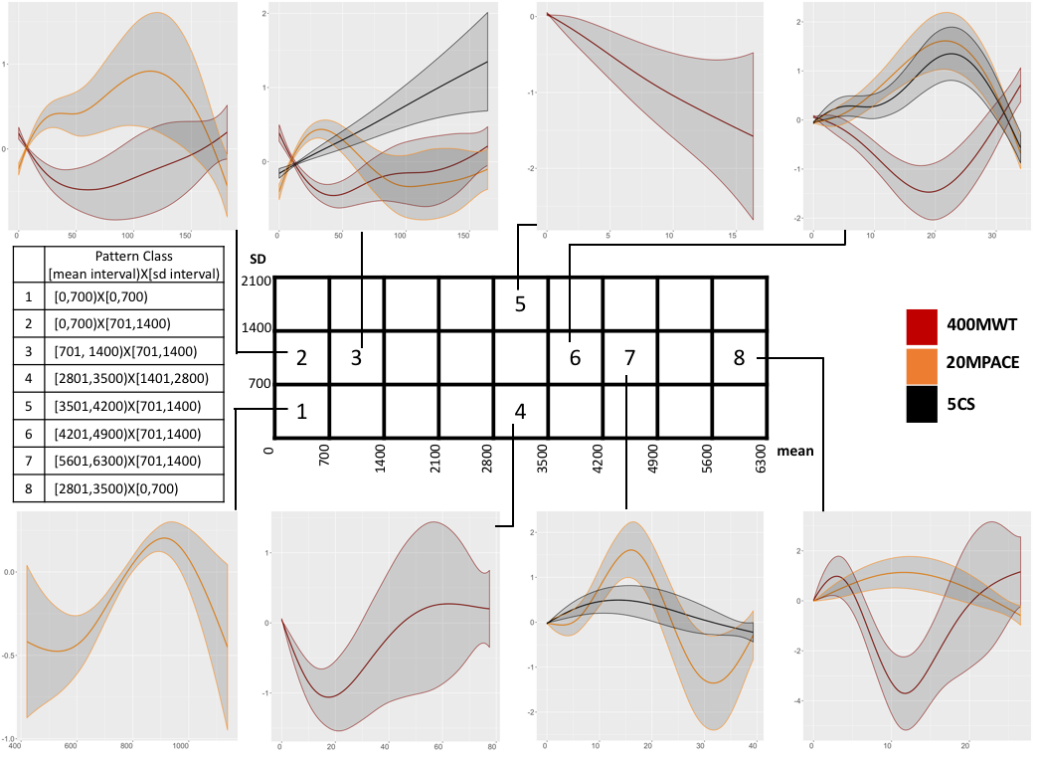
The grid boxes represent pattern classes labeled with the mean interval (x-axis) and the SD interval on the y-axis. 400MWT: 400-m walk test; 20MPACE: 20-m walk test; 5CSPACE: number of sit-to-stands per second measured over 5 repetitions.

GAMs fit smooth functions for each predictor in the model that additively contribute to the value of a latent variable. The model fitting algorithm [52] also estimates thresholds, whose values in relation to the latent variable computed from the smooth functions determine the ordered categorical response. Relationships between response and predictors in a GAM may be studied by plots of smoothers fitted by the GAMs. We studied predictors that were significant at *P*=.05 in the GAMs (Figure 6). Predictor-response relationships are shown by the smooth function plots arranged around the grid and linked to the corresponding predictor. We refer to a specific pattern class using the mean and SD interval pairs, as defined in the Methods section. The smooth function plots for pattern classes [0,700) X [701,1400) and [701,1400) X [701,1400) (numbered 2 and 3, respectively, on the mean-SD grid in Figure 6) show that up to 25 daily average minutes in these pattern classes were monotonically associated with improved response in the 400MWT and 20MPACE.

Most pattern classes with a higher mean and SD have low duration, resulting in fewer degrees of freedom for estimating the smooth functions at high values; this explains the wider confidence bands for the function estimates at higher values of daily average minutes. Inspection of a sample of instances from the class [701-1400) X [701-1400) revealed that long duration instances were typically spells of rest punctuated by frequent interruptions. A plausible explanation may be that such interruptions involve sit-stand transitions and, therefore, these instances are associated with the improved 5CSPACE response. Pattern classes with the mean interval [0-700) are not associated with the 5CSPACE response.

Pattern classes [2801,3500) X [0,700) and [2801,3500) X [1401,2100), numbered 4 and 5, respectively, are associated with the 400MWT response. The smooth function plots for these classes suggest that higher daily average minutes in both classes were associated with improved long-walk capacity. The association with increased completion times with >20 daily average minutes in the class [2801,3500) X [0,700) was due to instances comprising of mostly sedentary activity. Furthermore, infrequent occurrences of such instances resulted in wide estimate intervals for the smooth function.

**Table 2:** Gamma for generalized additive models evaluated on held-out data.

Predictors	Physical capacity measurement	Gamma
BMI^a^, age, sex, height, OA^b^ subcohort, function profile	400MWT^c^	0.62 (0.10)^d^
BMI, age, sex, height, OA subcohort, function profile	20MPACE^e^	0.53 (0.07)^d^
BMI, age, sex, height, OA subcohort, function profile	5CSPACE^f^	0.51 (0.04)^d^
BMI, age, sex, height, OA subcohort	400MWT	0.52
BMI, age, sex, height, OA subcohort	20MPACE	0.46
BMI, age, sex, height, OA subcohort	5CSPACE	0.47

^a^BMI: body mass index.

^b^OA: osteoarthritis.

^c^400MWT: 400-m walk test

^d^The values in parentheses indicate improvement over baseline performance without function profile predictors.

^e^20MPACE: the average pace in a 20-m walk test.

^f^5CSPACE: number of sit-to-stands per second measured over 5 repetitions.

Approximations of the distribution of activity counts in any given pattern class can be obtained by tail probability bounds. For example, use Chebyshev’s inequality, *P(|X – µ* *| > k* *σ) < (1/k*^*2*^*)* where *µ* and *σ* are the midinterval values of the mean and SD intervals, respectively, for a given pattern class. For the class [2801,3500) X [0,700), we obtained:

*P(|X –* 3150*| < k.* 350*) > (1 – 1/k*^*2*^*)* for *k*=1.8, implying that at least 70% of activity counts per minute were between 2520 and 3780. Thus, most of the activity in the class [2801,3500) X [0,700) was likely to be in the lower moderate-intensity range. Similarly, for the class [2801,3500) X [1401,2100), we noted that at least 70% of activity counts per minute were below 6300, indicating a mix of activity moderate and vigorous activity.

### Moderate-to-Vigorous Activity With Knee Malalignment

In the pattern class [3501,4200) X [701,1400), numbered 6 in the mean-SD grid of Figure 6, an increase in daily average minutes was monotonically associated with improved responses in all 3 capacity measures up to 20 minutes/day. However, an increase of >20 minutes was associated with a decline. Unlike the classes with low mean and SD, instances >20 minutes did not represent sedentary activity. A drop in physical function with increased time in moderate-to-vigorous activity is counterintuitive. To understand this finding, we reviewed patient-reported outcomes on the Physical Activity Scale for the Elderly (PASE). The PASE measures engagement in different kinds of daily activities related to leisure, household, and occupational work in the elderly [55]. In addition, we reviewed joint exam results reporting varus (bow-legged) and valgus (knock-kneed) alignments for the same subjects; this information is summarized in Table 3. It suggests that subjects with >20 daily average minutes in the pattern class [3501,4200) X [701,1400) had a higher prevalence and severity of knee deformity, higher time in the pattern class (minutes as well as frequency), and fewer sitting hours along with more walking hours per week.

**Table 3:** Knee deformity and PASE results of subjects with at least one instance of the pattern class [3501-4200) X [701-1400).

Subject results		Daily average minutes	
		≥20	<20
**Number of subjects with**		12	255
	Varus or valgus deformity in both knees	10	179
	Varus or valgus deformity in either knee	11	221
	Joint laxity (mild-severe) in either knee	8	109
Average number of days per week in activity		4.3	1.7
Average number of minutes per week in activity		161	43.4
Percentage with sitting hours <2, 2-4, >4 per day in last 7 days^a^		25, 50, 25	19, 55, 24
Percentage walking <1, 1-2, 2-4 h a day in last 7 days^a^		25, 33, 33	40, 40, 11

^a^Physical Activity Scale for the Elderly.

Studies have suggested that in subjects with knee malalignment or laxity, altered tibiofemoral loading could be responsible for biomechanical damage and OA progression [56-58]. A much debated view on the role of the quadriceps in OA is that the greater muscle strength in malaligned or lax knees increases the risk of OA progression [59,60]. If the relationship between the lower extremity strength and the risk of OA progression is confounded by the knee alignment status, a plausible explanation for the decreasing trend discussed above may be that regular investment in the pattern class [3501,4200) X [701,1400) promotes muscle strength but *advances* OA in subjects with malaligned knees. Though the current guidelines for knee OA management recommend muscle strengthening, our analysis highlights the need for a mechanistic investigation of greater power, given that muscle strength is a modifiable risk factor in OA.

## Discussion

### Principal Findings

To infer physical function from a daily activity trace, it is necessary to derive a representation that conveys information about the daily activity mix. We defined distinct segments from daily activity traces as instances of a set of pattern classes. Doing so transforms a sequence of activity counts into a sequence of pattern classes. Pattern classes provide an informative view of daily physical activity from the perspective of functional ability. Our approach of unsupervised segmentation and the subsequent definition of a set of pattern classes allows a function-based comparison among subjects without the overhead of obtaining annotated activity traces from subjects. This comparison is based on objective measurements and is, perhaps, the first effort to interpret functional outcomes based on pattern classes from free-living activity data, within a clinical research use case. Classifying physical function may be useful in several areas; for example, alternatives to outpatient physical therapy [61] are a topic of active research. Remote monitoring of physical function in daily living could allow rehabilitation programs to be evaluated in a site-less trial setting. We recognize that many clinical apps require a higher performance in physical function classification than obtained with our current models. Our results, however, suggest that this preliminary work may be advanced, potentially with higher resolution activity data.

### Limitations

There are 2 main limitations of our methods. First, the mean and SD are likely to be inadequate representations of the activity-generating processes, as they ignore temporal relationships between activity counts. Modeling class instances as subsequences generated by a random process have been proposed [62], and may improve the detection of pattern classes. Second, our approach ignores time ordering between pattern class instances in the daily activity profile. One way to address these limitations may be to learn within- and interclass relationships for a set of daily activity sequences, as a single Bayesian network. In addition, methods to reliably estimate the function profile from missing activity data are needed as nonadherence is a well-known issue in most health studies with wearable devices.

### Conclusions

An assessment of physical function based on the ability to perform routine tasks in daily life is desirable. Widely available wearable motion sensors can record daily activity objectively and unobtrusively. We have created an approach for deriving a function profile that represents time spent on various tasks encountered in daily living. Classifiers trained on the function profile were able to predict highest and lowest quartile results of clinically used physical capacity measures. We recovered associations between pattern classes and physical capacity measures, some of which corroborate prior OA research. The idea of representing physical function as a function profile derived from daily free-living activity may enable remote monitoring of patients’ physical function.

## Appendix

Multimedia Appendix 1 The osteoarthritis initiative dataset.

Multimedia Appendix 2 Quantitative & Ordinal Response Models.

Multimedia Appendix 3 Model Evaluation.

## Abbreviations

ADL: activities of daily living
GAM: Generalized Additive Models
OA: osteoarthritis
OAI: Osteoarthritis Initiative
PASE: Physical Activity Scale for the Elderly
WAM: wearable activity monitor
