# Supplementary material for: Inferring Physical Function From Wearable Activity Monitors: Analysis of Free-Living Activity Data From Patients With Knee Osteoarthritis
Source: JMIR Mhealth Uhealth. 2018 Dec 18;6(12):e11315. doi: 10.2196/11315 (PMC6315255; doi:10.2196/11315)
Supplement: Multimedia Appendix 1 [file mhealth_v6i12e11315_app1.pdf]

# Multimedia Appendix 1: The osteoarthritis initiative (OAI) dataset

The OAI dataset consist of historical clinical evaluation data, radiological images and a biospecimen repository from 4796 subjects. The subjects were enrolled between February 2004 and May 2006 and consisted of roughly equal number of men and women, aged between 45 – 79 years. Of these 2,712 were invited to participate in a physical activity study in the fourth year of the study and 2127 consented. 2001 subjects contributed activity data from one or more valid monitoring days.

**Progression subcohort**: Subjects with at least one symptomatic tibiofemoral knee (pain, aching or stiffness on most days for at least a month and a Kellgren & Lawrence score >=2) at baseline were eligible for the Progression subcohort.

**Incidence subcohort**: Incident symptomatic knee OA was defined as absence of the symptoms as defined for the progression cohort above, "frequent" knee symptoms and definite tibiofemoral osteophytes in the symptomatic knee.

Table 1 gives key demographic attributes of the subjects that participated in the physical activity study.

Table 1: Key Demographic attributes

| | |
|---|---|
| Cohort size | 2001 |
| Incidence subcohort | 1490 |
| Progression subcohort | 505 |
| Control subcohort | 6 |
| Elective knee replacement surgery before date of study | 63 |
| Age (mean, sd) | (65.08, 9.09) |
| Gender (% male) | 44.53 |
| BMI (mean, sd) | (28.52, 4.87) |
| Mean Comorbidity Index<br>(Modified Charleston Deyo) | 0.52 |
| Median physical activity days | 7 |

**Baseline physical capacity measurements**. Shown below are the baseline distributions of the 400m walk test, the 20m walk test (pace) and the 5 sit-stand test measurements on the subjects in the training partition.

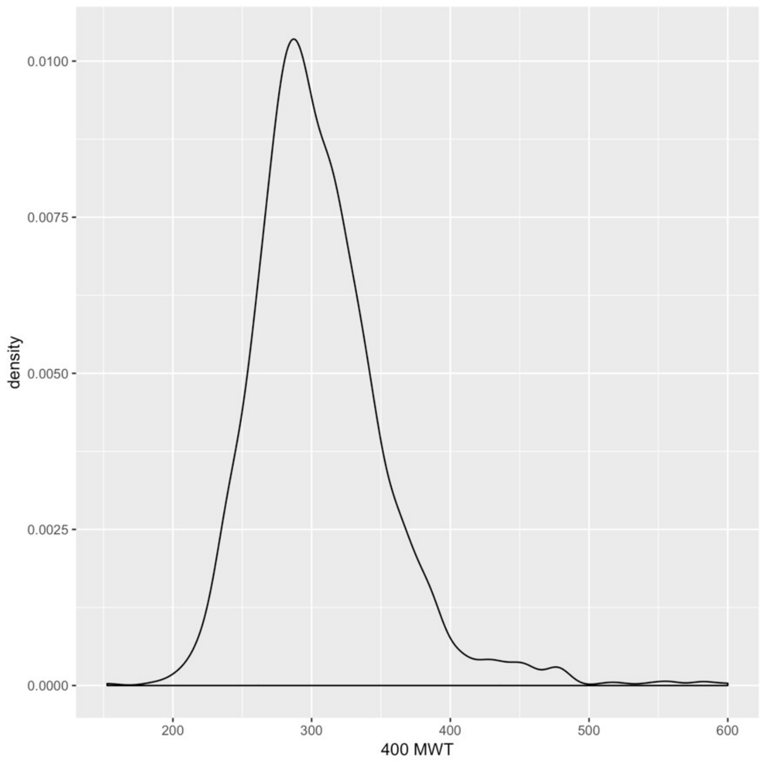
0.0100
0.0075
0.0050
0.0025
0.0000
density
200
300
400
500
600
400 MWT

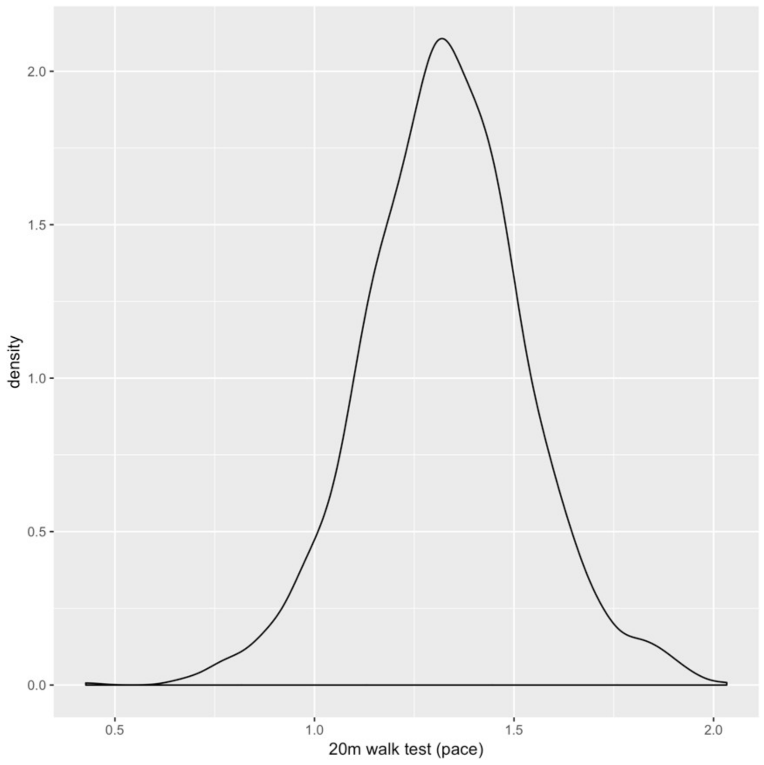
2.0
1.5
1.0
0.5
0.0
density
0.5
1.0
1.5
2.0
20m walk test (pace)

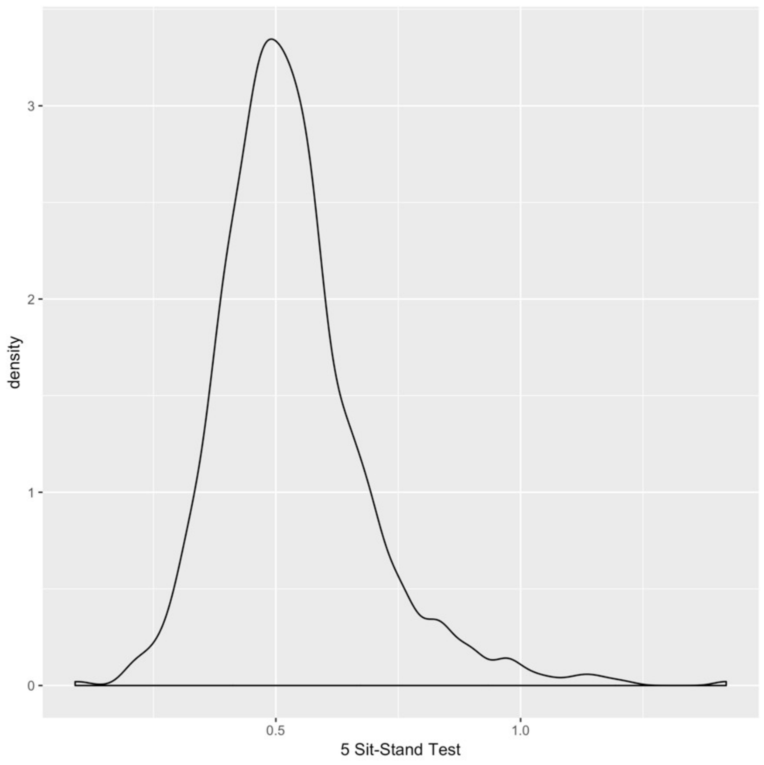

density
3
2
1
0
0.5
1.0
5 Sit-Stand Test
