# Supplementary material for: Inferring Physical Function From Wearable Activity Monitors: Analysis of Free-Living Activity Data From Patients With Knee Osteoarthritis
Source: JMIR Mhealth Uhealth. 2018 Dec 18;6(12):e11315. doi: 10.2196/11315 (PMC6315255; doi:10.2196/11315)
Supplement: Multimedia Appendix 2 [file mhealth_v6i12e11315_app2.pdf]

Multimedia Appendix 2: Quantitative & Ordinal Response Models

Quantitative Response Models

Our pattern classes populated a sparse feature space, with most instances occurring in the low mean and low SD regions. A popular technique for dealing with this problem is to use a non-smooth loss function[1] which allows some coefficients to be shrunk to zero. The R^2 for the fits and the mean squared error computed on the held out data for each LASSO regression model and different interval sizes is summarized in the table 1 below (based on a 20% held-out partition).

Table 1. R^2 and mean squared error for quantitative regression models

Interval size (I_{mean}, I_{SD})	400MTIM		20MPACE		5CSPACE		Iso Ext		Iso Flex	
	R^2	mse	R^2	mse	R^2	mse	R^2	mse	R^2	mse
(200,200)	0.262	2416.908	0.220	0.039	0.084	0.020	0.326	14.687	0.313	3.294
(400,400)	0.245	2416.000	0.227	0.038	0.088	0.020	0.339	14.569	0.340	3.160
(600,600)	0.253	2386.814	0.204	0.039	0.067	0.020	0.345	14.352	0.313	3.294
(800,800)	0.237	2456.437	0.218	0.038	0.109	0.019	0.337	14.675	0.320	3.255
(1000,1000)	0.255	2390.076	0.214	0.038	0.105	0.019	0.337	14.566	0.326	3.222
Best RMSE		48.855		0.195		0.138		3.788		1.778

The percentage of variance explained by the linear regression is low for all objective measures. The sensitivity of similar physical performance measures has been studied in clinical research within several medical specialties. For instance, in patients with hip OA, the minimum clinically important difference in the result of a 30 second chair stand test is 2[2]. Similarly, the minimal detectable change in the 6 minute walk test in patients undergoing a hip or knee arthroplasty has been reported as 61.34m[3]. While it is not clear what the minimum significant change values for the 400MWT in subjects with knee OA might be, it has been reported that an additional minute in completing the 400 MWT corresponds to a higher odds (OR 1.6, 95% CI 1.04-2.45) of developing incident disability in older adults with functional limitations[4]. The lowest root mean square errors (Best RMSE) for each objective measure over all interval sizes listed in table 2 indicate that the prediction errors from our models frequently exceed the threshold of the clinically relevant minimum change.

However, the dispersion of the fitted values suggests that we may be able to predict the correct quantile. This is illustrated in figure 1A that shows a scatter plot of the predicted and observed values of the 400MWT from the held out data for (for bounding region of size 1000,1000). Predicting ordered category values for the held-out data, on the basis of the 25th and 75th percentile in the fitted values gives a gamma = 0.59. Figure 1B shows the AUC of high/low classifiers (with respect to the median) for each objective measure, based on an L1 penalized loss function.

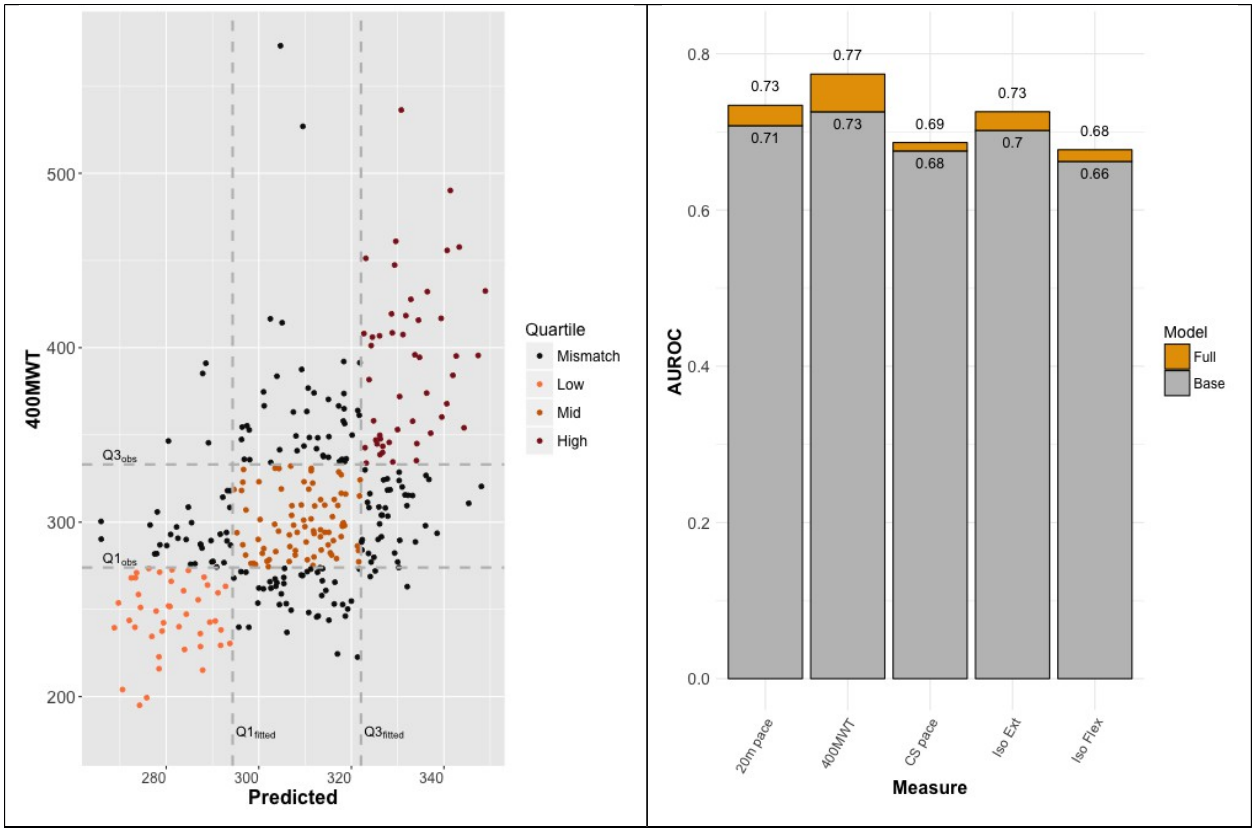

Figure 1. A) Predicting ordered categorical values based on quartiles. The colored points represent held out observations for which the predicted and observed categories match. Categories were assigned using the 25th and 75th percentile values in the observed and fitted values respectively. Black points represent incorrect predictions. B) AUC on high/low prediction tasks on held out data. The orange colored bar segments represent the contribution of the activity profile features.

Goodman Kruskal Gamma

The predicted ordinal responses may differ from the truth by degrees (for example, a predicted response 3 against a truth value of 1 is worse than a predicted response 2), quantifying the performance of ordinal response models may be done via the ratio of concordance probability to the discordance probability. Known as the Goodman Kruskal Gamma statistic, it represents the extent to which a monotone trend association exists between the predicted and true values. Formally, the Gamma statistic is defined as

$$\gamma = \frac{\Pi_c - \Pi_d}{\Pi_c + \Pi_d} \quad \text{where,}$$

$$\Pi_c = 2\sum_i \sum_j \pi_{ij}\left(\sum_{h>i} \sum_{k>j} \pi_{hk}\right), \Pi_d = 2\sum_i \sum_j \pi_{ij}\left(\sum_{h>i} \sum_{k<j} \pi_{hk}\right) \quad \text{and,}$$

π_{ij} is the joint probability distribution of the fitted and true categories

References

1. Tibshirani R. Regression Shrinkage and Selection via the Lasso. J R Stat Soc Ser B. WileyRoyal Statistical Society; 1996;58:267–288.
2. Wright AA, Cook CE, Baxter GD, Dockerty JD, Abbott JH. A Comparison of 3 Methodological Approaches to Defining Major Clinically Important Improvement of 4 Performance Measures in Patients With Hip Osteoarthritis. J Orthop Sport Phys Ther. 2011;41(5):319–327. PMID: 21335930
3. Kennedy DM, Stratford PW, Wessel J, Gollish JD, Penney D. Assessing stability and change of four performance measures: a longitudinal study evaluating outcome following total hip and knee arthroplasty. BMC Musculoskelet Disord. 2005;6(1):3. PMID: 15679884
4. Chang M, Cohen-Mansfield J, Ferrucci L, Leveille S, Volpato S, De Rekeneire N, et al. Incidence of Loss of Ability to Walk 400 Meters in a Functionally Limited Older Population. J Am Geriatr Soc. Blackwell Science Inc; 2004;52(12):2094–2098.
5. Archer KJ. glmpathcr : An R Package for Ordinal Response Prediction in High-dimensional Data Settings. (2).
