# Supplementary material for: Inferring Physical Function From Wearable Activity Monitors: Analysis of Free-Living Activity Data From Patients With Knee Osteoarthritis
Source: JMIR Mhealth Uhealth. 2018 Dec 18;6(12):e11315. doi: 10.2196/11315 (PMC6315255; doi:10.2196/11315)
Supplement: Multimedia Appendix 3 [file mhealth_v6i12e11315_app3.pdf]

Multimedia Appendix 3: Model evaluation

AUC 1 and 3 refer to the performance in the 1 versus rest and 3 versus rest classification tasks, respectively. Including the activity profile improved the held out AUC by 1% – 4% and Gamma by 4%-11%, compared to classifiers in which activity profile was excluded from the predictors, with higher improvement in classification of walking performance (400MWT and 20MPACE). 95% confidence intervals for the improvements were computed using a bootstrap procedure. The lowest quartile classification in 20MPACE and 5CSPACE improved the least with the inclusion of the function profile. At the optimal cutoff point, the AUCs for the 400MWT function profile based on an interval size of (700,700) had sensitivity = 0.72 and specificity = 0.71 for the lowest quartile. In the highest quartile, sensitivity was 0.67 and specificity was 0.74. The optimal cut-off points for classifying gait speed in the lowest quartile for 20MPACE with the same interval size were 0.76 for sensitivity and 0.68 for specificity.

Predictors	Physical Capacity measurement	Gamma	Gamma improvement (95% CI)	AUC1	AUC3
BMI, age, sex, height, OA subcohort, function profile	400MWT	0.63	0.11 (0.06, 0.14)	0.75	0.77
BMI, age, sex, height, OA subcohort, function profile	20MPACE	0.53	0.07 (0.03, 0.12)	0.79	0.65
BMI, age, sex, height, OA subcohort, function profile	5CSPACE	0.51	0.04 (0.01, 0.12)	0.73	0.73
BMI, age, sex, height, OA subcohort	400MWT	0.52	-	0.74	0.73
BMI, age, sex, height, OA subcohort	20MPACE	0.46	-	0.74	0.66
BMI, age, sex, height, OA subcohort	5CSPACE	0.47	-	0.72	0.72
